# The power-proportion method for intracranial volume correction in volumetric imaging analysis

**DOI:** 10.3389/fnins.2014.00356

**Published:** 2014-11-06

**Authors:** Dawei Liu, Hans J. Johnson, Jeffrey D. Long, Vincent A. Magnotta, Jane S. Paulsen

**Affiliations:** ^1^Department of Psychiatry, Carver College of Medicine, University of IowaIowa City, IA, USA; ^2^Department of Electrical and Computer Engineering, College of Engineering, University of IowaIowa City, IA, USA; ^3^Department of Biomedical Engineering, College of Engineering, University of IowaIowa City, IA, USA; ^4^Department of Biostatistics, College of Public Health, University of IowaIowa City, IA, USA; ^5^Department of Radiology, Carver College of Medicine, University of IowaIowa City, IA, USA; ^6^Department of Neurology, Carver College of Medicine, University of IowaIowa City, IA, USA; ^7^Department of Psychology, University of IowaIowa City, IA, USA

**Keywords:** magnetic resonance imaging, intracranial volume, nonlinear model, power function, power-proportion correction

## Abstract

In volumetric brain imaging analysis, volumes of brain structures are typically assumed to be proportional or linearly related to intracranial volume (*ICV*). However, evidence abounds that many brain structures have power law relationships with *ICV*. To take this relationship into account in volumetric imaging analysis, we propose a power law based method—the power-proportion method—for *ICV* correction. The performance of the new method is demonstrated using data from the PREDICT-HD study.

## Introduction

In magnetic resonance imaging (MRI) studies of the human brain, volumetric analysis of anatomical brain regions plays an important role in studying disease states. Regional volumetric changes are linked to various brain diseases. For example, reduced basal ganglia volume is a hallmark of Huntington disease (Aylward et al., 1997, 2012). Reduced hippocampal volume has been shown to be predictive of memory loss (Ystad et al., 2009). Atrophy of the frontal and temporal lobes are associated with frontotemporal dementia (Hodges et al., 1999; Marra et al., 2007); and patients with autism tend to have an enlarged amygdala (Schumann et al., 2009). An accurate evaluation of the volumetric difference in brain regions between groups of individuals is not only a critical step toward a better understanding of brain related disorders, but also vital for the discovery of neuroimaging biomarkers for these disorders.

It is a challenging task to precisely quantify the volumetric differences in a brain region of interest between individuals. The major difficulty lies in the fact that individuals vary in overall head size, which naturally leads to variations in the regional volume of interest (*VOI*). Therefore, when comparing a *VOI* between groups of people, it is crucial that the confounding effect of head size be controlled. In brain imaging, intracranial volume (*ICV*) is a popular measure of head size, thus the control of the confounding effect of head size is often carried out through *ICV* correction.

There are a couple of widely used methods for *ICV* correction in neuroimaging research. One popular practice is to divide a raw *VOI* by *ICV* and use the *VOI*-to-*ICV* ratio in statistical analysis (Hubbard and Anderson, 1981; Mathalon et al., 1993; Sanfilipo et al., 2004; Kruggel, 2006; O'Brien et al., 2011). The method is called the “proportion method.” The implicit assumption underlying this method is that a *VOI* is proportional to *ICV*. Another popular method is the analysis of covariance (ANCOVA) method (Andreasen et al., 1986; Mathalon et al., 1993; Sanfilipo et al., 2004; O'Brien et al., 2011), in which *ICV* is included as a covariate in a regression model whose dependent variable is a *VOI*. It has been shown to perform better than the proportion method (Sanfilipo et al., 2004; O'Brien et al., 2011). The ANCOVA method relaxes the proportionality assumption on the relationship between a *VOI* and *ICV*, but assumes that *VOI* is linearly related to *ICV*.

However, evidence abounds that most brain structures relationships to *ICV* are neither proportional nor linear. Studies have shown that the relationships between many *VOI*s and *ICV* follow the power law principle (Zhang and Sejnowski, 2000; Lüders et al., 2002; Im et al., 2008; Barnes et al., 2010), that is, a *VOI* is related to *ICV* through the relationship *VOI* = α * *ICV*^β^. For example, in the study of Im et al. (2008), the authors investigated the relationships between *ICV* and lobar cortical volume based on a sample of 148 normal individuals and found the relationships follow power law, and the scaling exponents for four lobes are between 0.836 and 0.901. In an article by Barnes et al. (2010), they studied a number of brain *VOI*s, including gray and white matter, lateral ventricles and some subcortical structures, and concluded that all the *VOI*s, except for the lateral ventricles, were related to *ICV* according to the power law, with the scaling exponent less than one. From a different perspective, Zhang and Sejnowski (2000) reported the relationships between gray and white matter volumes in 59 species follow the power law. Lüders et al. (2002) also illustrated the power law phenomenon between brain size and gray matter volume. These studies suggested that the power law may be a fundamental principle governing many biological processes.

With more and more evidence showing that the power law principle is behind the relationships between brain *VOI*s and *ICV*, an effective *ICV* correction method should utilize the power law relationship. In this paper, we propose a power law based *ICV* correction method—the “power-proportion method.”

## Materials and methods

### Ethics statement

The research protocol was approved by the University of Iowa Institutional Review Board, and all participants gave written informed consent and were treated in accordance with the principles expressed in the Declaration of Helsinki.

### Participants

PREDICT-HD is a multi-site longitudinal observational study of individuals who were known to have the genetic risk for Huntington diseased (HD) (Paulsen et al., 2006, 2008). There are both healthy controls and at-risk participants in PREDICT-HD, but only healthy controls were included in the analysis. To eliminate the nuisance effects of different scanner manufacturers and field strengths, only participants who were scanned on the 1.5T GE Signa MR scanner were included. All data came from the baseline visit.

There were 141 eligible participants in our study sample, with 92 of them being female. Descriptive statistics for the sample are summarized in Table 1. The males were on average 2.1 years younger than the females, but the difference was not statistically significant. There was a significant difference in *ICV* between males and females, with males being on average 198.12 cc larger than females.

**Table 1:** **Demographic information of the sample**.

	**Male**	**Female**	***p*-value**
Controls	49	92	n/a
Mean age (*SD*)	42.68 (10.41)	44.78 (10.41)	0.2730
Mean *ICV* (cc) (*SD*)	1712.01 (138.54)	1513.88 (122.12)	<0.0001

*ICV, intracranial volume*.

### MRI acquisition

All MRI images were obtained on a 1.5T GE Signa MR scanner. Three different sequences were acquired for each participant: T1- and T2-weighted images as well as proton-density (PD) weighted images. The T1-weighted images were collected using an axial 3D volumetric spoiled-gradient echo sequence, with a flip angle of 40°, *TE* = 5 ms, *TR* = 24 ms, *FOV* = 24 cm, 124 coronal slices at 1.5 mm/slice, matrix dimension 256 × 192 and NEX = 2. Only the T1 images were used in the image processing and analysis.

### Image analysis

Imaging analysis was performed using FreeSurfer (version 5.0, http://surfer.nmr.mgh.harvard.edu), an automated surface reconstruction and tissue classification and segmentation software. Each participant's MRI scan was analyzed in original space using the standard FreeSurfer analysis pipeline. Briefly, processing included removal of non-brain tissue by a hybrid watershed/surface deformation procedure (Ségonne et al., 2004), subcortical structures were segmented (Fischl et al., 2002), and further intensity normalization was conducted. This was followed by white-matter segmentation, tessellation of the gray-white matter boundary, and automated topology correction (Fischl et al., 2001). Then surface deformation following intensity gradients optimally places the gray/white and gray/cerebrospinal fluid (CSF) borders at the location where the greatest shift in intensity defines the transition to the other tissue class (Fischl et al., 2001). Once the cortical models were complete, additional data processing and analysis were performed with deformable procedures, including parcellation of the cerebral cortex into 34 (Desikan et al., 2006) or 74 (Destrieux et al., 2010) conventional gyral- and sulcal-based neuroanatomical regions in each hemisphere. In automatic subcortical segmentation, each voxel in the normalized brain volume was assigned one of 37 labels. The *ICV* was estimated using the method described by Buckner et al. (2004). Previous studies have shown that FreeSurfer-derived volumetric measures of *ICV* (Buckner et al., 2004; Jovicich et al., 2009), cortical structures (Wonderlick et al., 2009) and subcortical structures (Jovicich et al., 2009; Wonderlick et al., 2009) all have high reliabilities.

### Empirical evidence of the non-proportionality between *VOI*s and *ICV*

**Figure 1:**
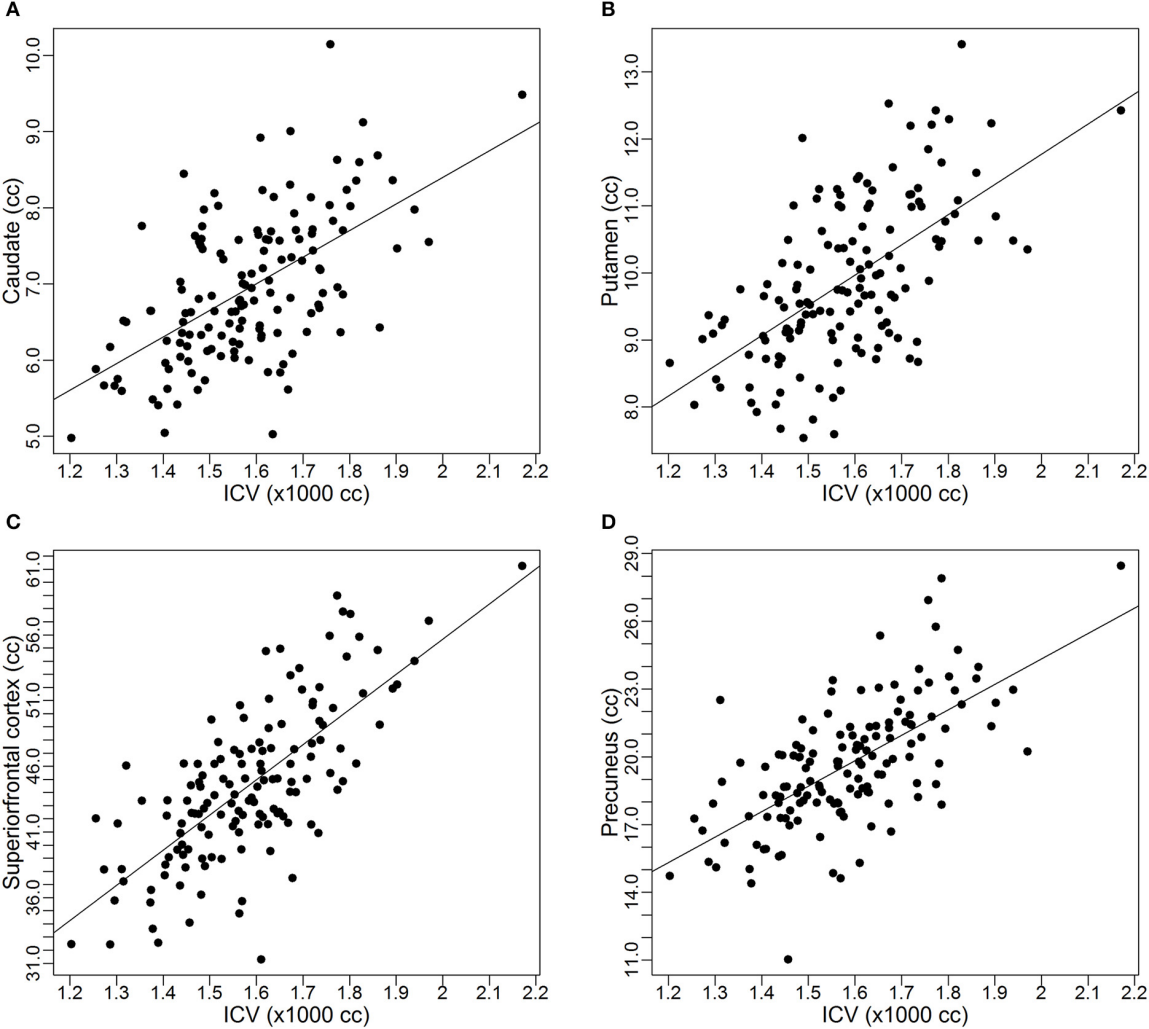
**Scatterplots and linear regression lines of the raw volumes vs. intracranial volume. (A)** Caudate; **(B)** putamen; **(C)** superior frontal cortex; **(D)** precuneus. *ICV*, intracranial volume.

**Figure 2:**
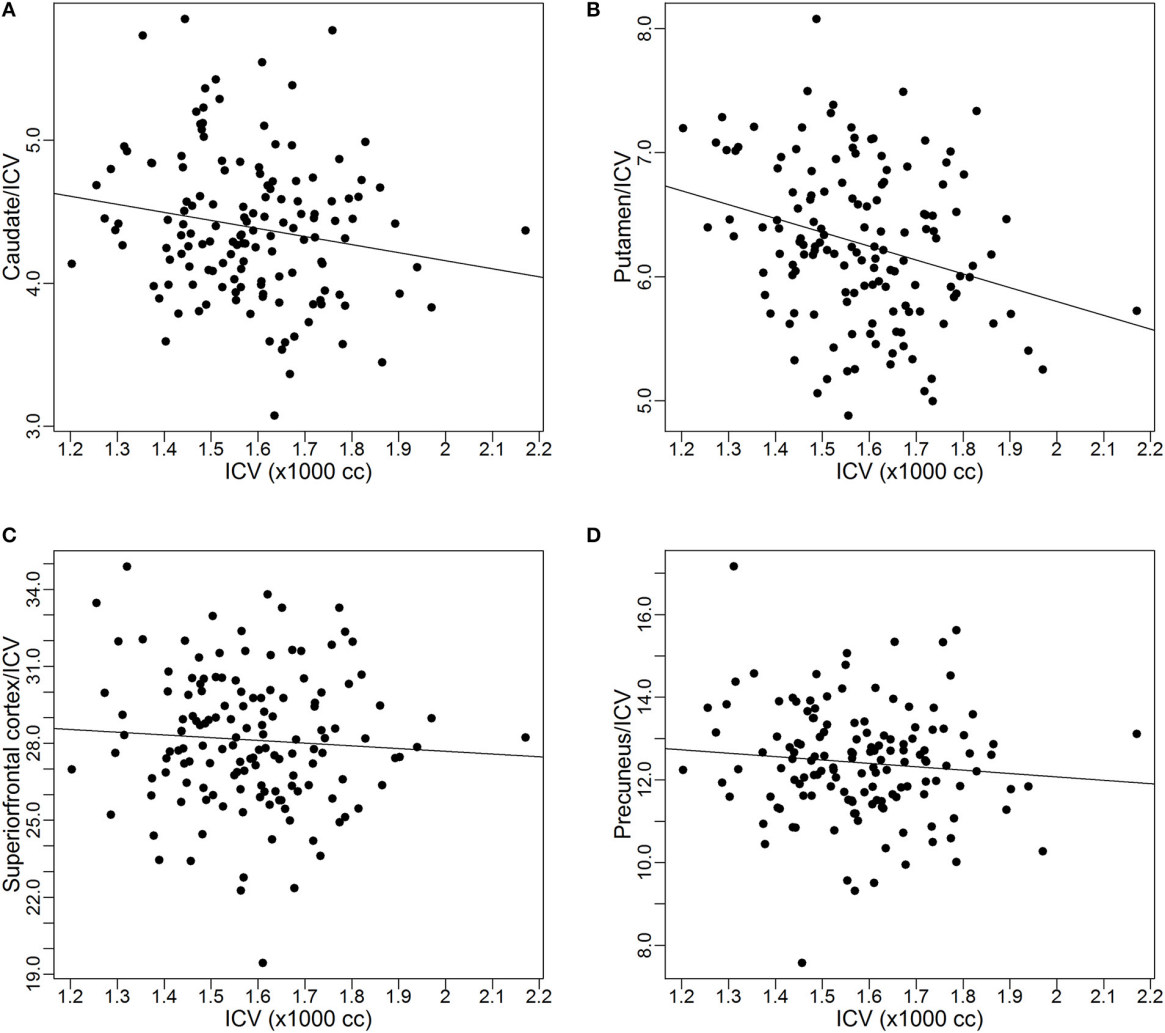
**Scatterplots and linear regression lines of the proportion corrected volumes vs. intracranial volume. (A)** Caudate; **(B)** putamen; **(C)** superior frontal cortex; **(D)** precuneus. *ICV*, intracranial volume.

As discussed earlier, some studies already reported the proportional relationships between *VOI*s and *ICV* may not always hold. In this subsection, we use data from the PREDICT-HD study to further demonstrate the non-proportionality between *VOI*s and *ICV*. Four brain structures are used as examples: caudate, putamen, superior frontal cortex, and precuneus. Figure 1 shows the scatter plots of these four *VOI*s vs. *ICV*. All plots show that each *VOI* is positively correlated with *ICV*, which verifies that these *VOI*s increase with head size. After dividing each *VOI* by *ICV*, which is the proportion method of *ICV* correction, the scatterplots of *VOI*-to-*ICV* ratios vs. *ICV* are shown in Figure 2. All plots show a negative trend, with varying degrees, between the proportion-corrected volume and *ICV*: The larger the *ICV*, the smaller the corrected volume, and vice versa. If each *VOI* is proportional to *ICV*, there should be no trend in any plot. These plots demonstrate that a brain *VOI* is in general not proportional to *ICV*.

### The power-proportion correction method

In this subsection, we present the power-proportion method that employs the power law principle to correct for individual differences in *ICV*. As was stated earlier, the power law principle stipulates that a *VOI* is related to *ICV* through the relationship:

$$VOI=\alpha ICV^{\beta },$$

where α is a constant and β is the scaling exponent of the power function. Both α and β can depend on the *VOI*. When β = 1, the *VOI* is in proportion to *ICV*. When β < 1, for a certain percentage increase in *ICV*, the percentage increase in *VOI* will be smaller; when β > 1, for a certain percentage increase in *ICV*, the percentage increase in *VOI* will be larger.

For the estimation of α and β, the following nonlinear model is utilized

(1) $$VOI=\alpha ICV^{\beta }+\varepsilon ,$$

where ε is the random noise that is assumed to follow an independent and identical (iid) normal distribution with mean 0 and some variance σ^2^, that is ε ~ N(0, σ^2^). It is noted that in this model formulation, the random noise is assumed to have an additive effect on the *VOI*. The implication of the additive assumption is that the variance of the noise is about constant across *ICV* values. This assumption is supported by the empirical evidence provided by our data. For example, in Figure 1, although each *VOI* is increasing with *ICV*, the variability is about the same at different values of *ICV*.

The estimate of β, denoted by *b*, is obtained from fitting Model 1 using sample data. Then *b* can be used in the power-proportion correction of *ICV* using the following formula:

(2) $$VOI_{PPC}=\frac{VOI}{ICV^{b}},$$

where *VOI_PPC_* denotes the power-proportion corrected *VOI*. Note that this correction formula takes a similar form as the proportion method. The difference lies in the denominator. In the proportion method, the exponent of *ICV* is fixed to be *b* = 1 for all regions and all studies, whereas in the power-proportion correction method, *ICV* is a power function with the exponent being estimated from the study sample. If *b* is equal to 1, then the nonlinear corrected *VOI* is equivalent to the traditional proportion method. As *b* diverges away from 1, then the power-proportion corrected *VOI* diverges from the proportion method.

## Results

The power-proportion method as described in the previous subsection was applied to data from the PREDICT-HD study. Analysis was conducted on 34 cortical regions based on the parcellation scheme of Desikan et al. (2006), the CSF and eight subcortical regions from the FreeSurfer output, and the results were tabulated.

Before presenting results from all these 43 *VOI*s, estimates for the four ROIs that were used as examples in the previous subsection were extracted: caudate, putamen, superior frontal cortex, and precuneus. Table 2 shows the estimates of the exponent parameter β and their standard errors (in parentheses) from the fitting of Model 1. The table shows the estimated exponents for all four *VOI*s are smaller than 1. The β estimates for two of the *VOI*s (caudate and putamen) are significantly smaller than 1 (95% confidence interval does not contain 1), which suggests that the proportional relationship between these *VOI*s and *ICV* does not hold and explains why the proportion-corrected *VOI*s are strongly negatively correlated with *ICV*, as illustrated in Figure 2. The estimates for prefrontal cortex and precuneus are not significantly different from 1 (95% confidence interval contains 1), which indicates the proportional relationship approximately holds between these *VOI*s and *ICV*. This result also demonstrates the proposed power-proportion method includes the proportion method as a special case: When the proportional relationship between a *VOI* and *ICV* holds, the former reduces to the latter.

**Table 2:** **Regression estimates and their standard errors (*SE*) of the exponent of the power function of intracranial volume for each volume of interest**.

***VOI***	**Estimate of β (*SE*)**	**95% CI**
Caudate	0.80 (0.10)	(0.61, 0.99)
Putamen	0.72 (0.08)	(0.56, 0.88)
Superior frontal cortex	0.96 (0.08)	(0.81, 1.11)
Precuneus	0.91 (0.09)	(0.74, 1.09)

*VOI, volume of interest; CI, confidence interval*.

**Figure 3:**
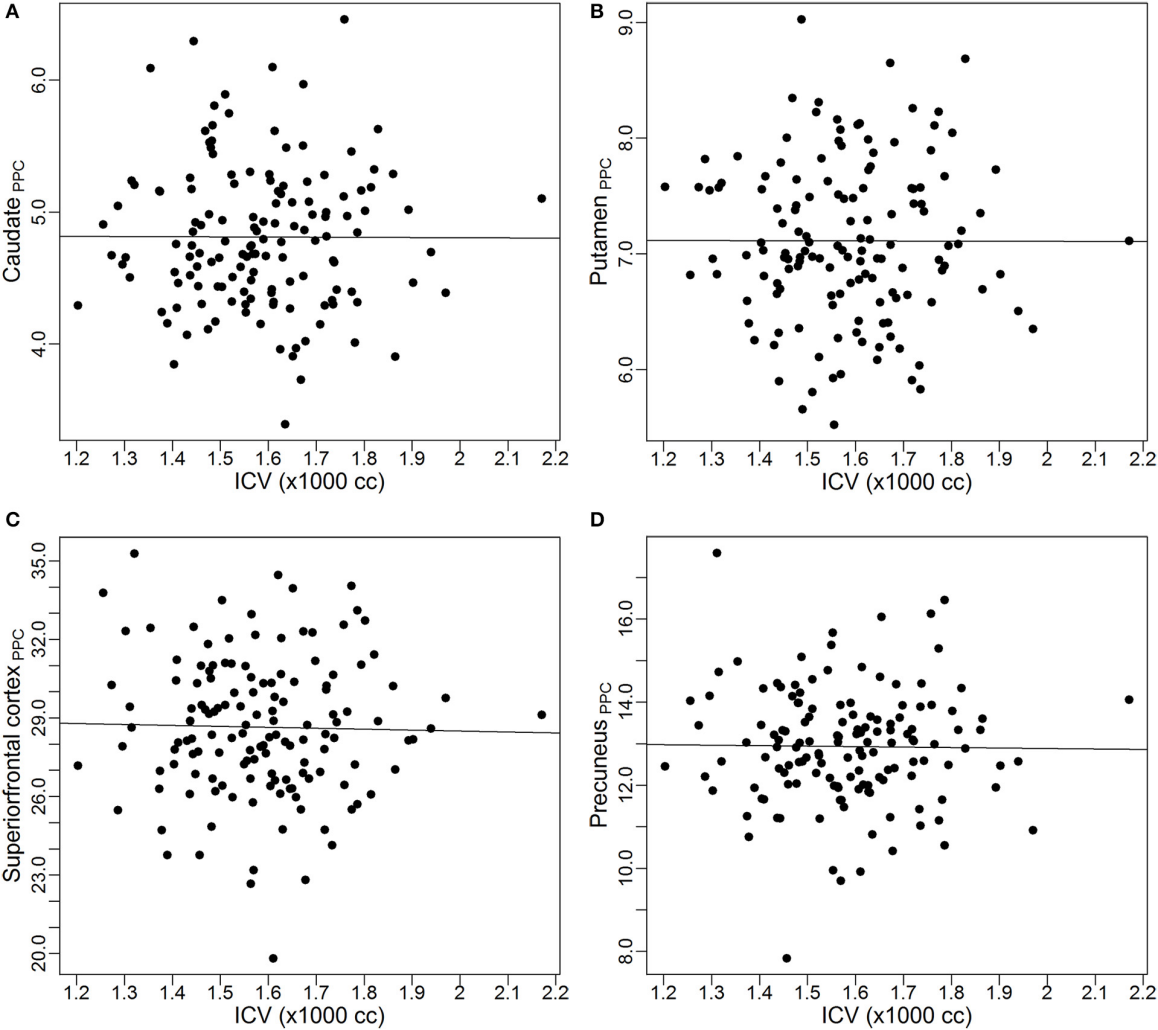
**Scatterplots and linear regression lines of the power-proportion corrected volumes vs. intracranial volume. (A)** Caudate; **(B)** putamen; **(C)** superior frontal cortex; **(D)** precuneus. *ICV*, intracranial volume. The subscript “PPC” in the label of y-axis represents “power-proportion correction.”

To visually inspect the performance of the power-proportion method in removing the confounding effect of *ICV*, scatterplots of *VOI_PPC_* vs. *ICV* for the four *VOI*s are presented in Figure 3. The straight line in each plot represents the regression line between *VOI_PPC_* and *ICV*. The plots show each line is parallel to the x-axis, which indicates after the power-proportion correction, the *ICV*-corrected *VOI*s are uncorrelated with *ICV*. This is in contrast to the plots in Figure 2 based on the proportion correction method, where almost all regression lines have a negative trend.

Tables 3, 4 summarized the estimates for nine subcortical *VOI*s (including CSF) and 34 cortical *VOI*s, respectively. From these tables it is clear that the estimated exponent parameter β for many *VOI*s are significantly less than 1, such as the caudate, putamen, amygdala, hippocampus, pallidum, caudal anterior cingulate, cuneus, temporal pole, among others. This indicates when *ICV* increases by a certain percentage, these *VOI*s do not increase by the same percentage. Rather, they increase at a lower rate.

**Table 3:** **Regression estimates of the exponent of the power function of intracranial volume for subcortical volumes of interest**.

***VOI***	**Estimate of β**	***SE***	**Lower 95% confidence limit**	**Upper 95% confidence limit**
Caudate	0.80	0.10	0.60	1.00
Putamen	0.72	0.08	0.56	0.88
Amygdala	0.77	0.08	0.61	0.93
Hippocampus	0.62	0.06	0.50	0.74
Pallidum	0.80	0.08	0.64	0.96
Accumbens	0.95	0.11	0.73	1.17
Thalamus	0.89	0.07	0.75	1.03
Lateral ventricle	1.57	0.41	0.77	2.37
CSF	0.66	0.19	0.29	1.03

*VOI, volume of interest; CSF, cerebrospinal fluid*.

**Table 4:** **Regression estimates of the exponent of the power function of intracranial volume for cortical volumes of interest**.

**Volume of Interest (*VOI*)**	**Estimate of β**	***SE***	**Lower 95% confidence limit**	**Upper 95% confidence limit**
Bankssts	0.86	0.12	0.62	1.10
Caudalanteriorcingulate	0.69	0.14	0.42	0.96
Caudalmiddlefrontal	0.93	0.11	0.71	1.15
Cuneus	0.52	0.12	0.28	0.76
Entorhinal	1.04	0.12	0.80	1.28
Fusiform	0.80	0.08	0.64	0.96
Inferiorparietal	0.80	0.10	0.60	1.00
Inferiortemporal	1.06	0.09	0.88	1.24
Isthmuscingulate	0.88	0.10	0.68	1.08
Lateraloccipital	0.67	0.08	0.51	0.83
Lateralorbitofrontal	0.89	0.07	0.75	1.03
Lingual	0.57	0.11	0.35	0.79
Medialorbitofrontal	0.94	0.08	0.78	1.10
Middletemporal	1.00	0.09	0.82	1.18
Parahippocampal	0.61	0.10	0.41	0.81
Paracentral	0.71	0.10	0.51	0.91
Parsopercularis	1.04	0.12	0.80	1.28
Parsorbitalis	0.65	0.11	0.43	0.87
Parstriangularis	0.94	0.12	0.70	1.18
Pericalcarine	0.64	0.16	0.33	0.95
Postcentral	0.88	0.09	0.70	1.06
Posteriorcingulate	0.96	0.09	0.78	1.14
Precentral	0.73	0.08	0.57	0.89
Precuneus	0.91	0.09	0.73	1.09
Rostralanteriorcingulate	1.17	0.12	0.93	1.41
Rostralmiddlefrontal	1.04	0.10	0.84	1.24
Superiorfrontal	0.96	0.08	0.80	1.12
Superiorparietal	0.80	0.10	0.60	1.00
Superiortemporal	0.89	0.08	0.73	1.05
Supramarginal	0.94	0.09	0.76	1.12
Frontalpole	0.68	0.15	0.39	0.97
Temporalpole	0.52	0.11	0.30	0.74
Transversetemporal	0.81	0.13	0.56	1.06
Insula	1.06	0.07	0.92	1.20

**Figure 4:**
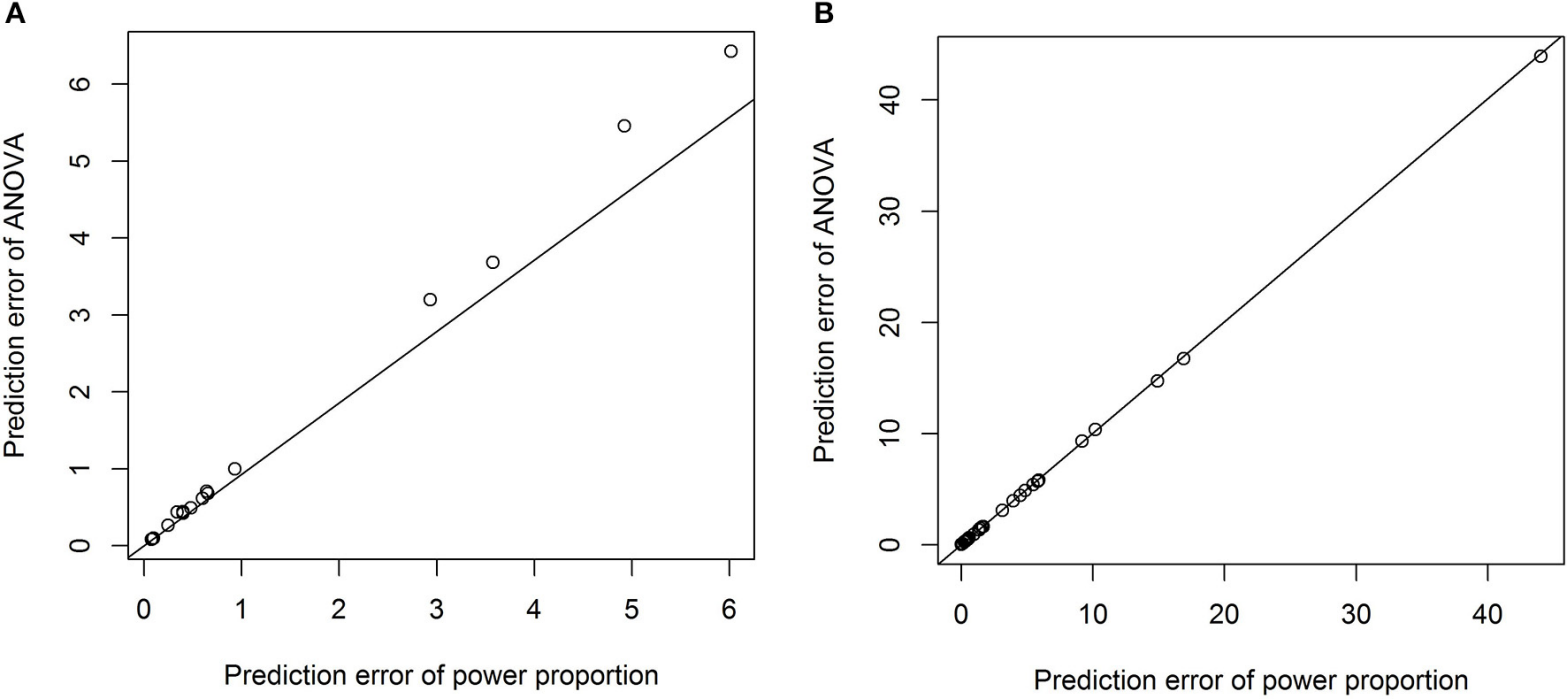
**Scatterplots of prediction errors based on the power-proportion method and the ANCOVA method. (A)** When the exponent parameter is significantly different from 1; **(B)** when the exponent parameter is not significantly different from 1.

To formally compare the performance of the power-proportion method and the ANCOVA method in describing the relationship between a *VOI* and *ICV*, leave-one-out cross-validation was conducted and the prediction error (sum of the squared differences between observed and predicted responses) was calculated. Scatterplots of prediction errors from the two methods are shown in Figure 4, where the straight line in each plot represents the line with 45° angle. From these plots it is clear that when the nonlinear relationship between a *VOI* and *ICV* is strong (the exponent of the power function is significantly different from 1), the prediction error based on the power-proportion method are smaller than that based on the ANCOVA method (plot A); whereas when the linear relationship between a *VOI* and *ICV* approximately holds, the prediction errors are very similar (plot B). This is not surprising as the power-proportion method includes ANCOVA method as a special case.

## Discussion

In volumetric comparison of a brain region between groups of individuals, the proportion method is often applied to correct for individual differences in head size. The underlying assumption of this method is that a *VOI* is proportional to *ICV*. Using examples from previous publications as well as from the PREDICT-HD study, we showed for many brain structures, their relationships to *ICV* are not proportional. Rather, the relationships follow the power law.

Many studies have explicitly (for example, Im et al., 2008) or implicitly (for example, Barnes et al., 2010) reported the power law phenomenon for different brain structures. Our own data also supported this finding. To take into account the power law relationship, we proposed the power-proportion method for *ICV* correction. Our analyses have shown the new method achieves better performance compared to the proportion method in that the new *ICV*-corrected *VOI* has a near-zero correlation with *ICV*. The new method has the potential to increase the likelihood of valid volumetric comparisons between groups by effectively removing the confounding effect of *ICV*.

**Figure 5:**
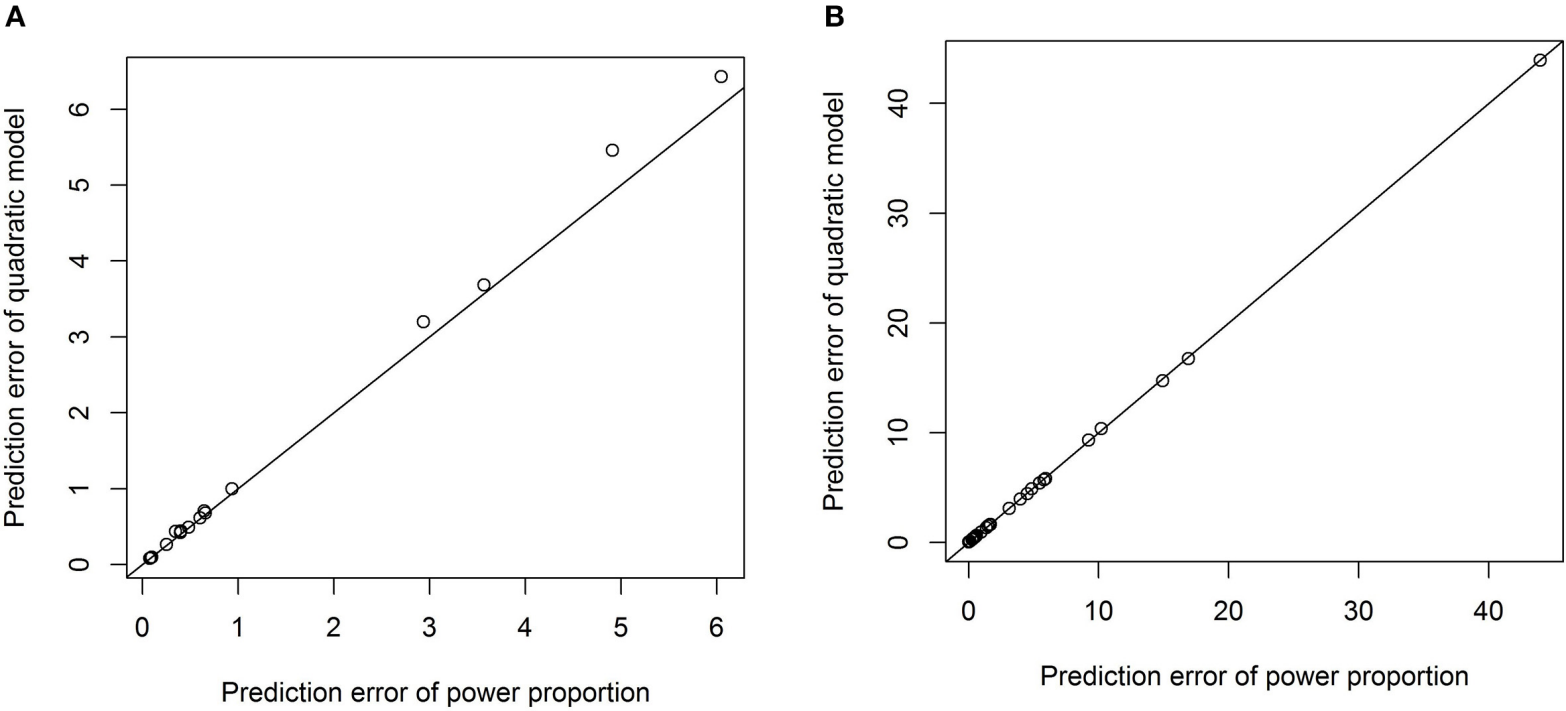
**Scatterplots of prediction errors based on the power-proportion method and the ANCOVA method with the quadratic term. (A)** When the exponent parameter is significantly different from 1; **(B)** when the exponent parameter is not significantly different from 1.

One advantage of our proposed method is that the exponential parameter of the power-law relation is estimated from the data rather than specified a priori. For example, we have tested the quadratic relation between each *VOI* and *ICV* and it turned out that the quadratic relationship is significant only for three out of forty three *VOI*s. The prediction errors from the model with the quadratic term and those from the power-proportion method are compared and summarized using scatterplots in Figure 5. Comparing Figure 5 to Figure 4, we can see that the prediction errors from the model with the quadratic term are reduced compared to the ones from the linear model, but they are still larger than those from the power-proportion method. This indicates the existence of nonlinear relationship between *VOI*s and *ICV* and highlights the importance of estimating rather pre-specifying the potential nonlinear relationship between a *VOI* and *ICV*.

We would like to point out that our proposed method also has close connection to the residual or ANCOVA method for *ICV* correction (Mathalon et al., 1993; Sanfilipo et al., 2004; O'Brien et al., 2011). If in the analysis of volumetric data, both the *VOI* and *ICV* are log-transformed and a linear regression is fitted based on the log-transformed data (which is the idea of residual or ANCOVA method), the modeling practice is similar to the nonlinear regression as presented in Model 1. However, there is a critical difference between these two modeling strategies. For the regression on log-transformed data, the underlying assumption for the effect of random noise is that it affects *VOI* in a multiplicative manner. A consequence of this assumption is that the variability of *VOI* increases with *ICV*. On the other hand, our nonlinear model formulation assumes the additive effect of random noise. The implication of this assumption is that the variability of the *VOI* is constant across values of *ICV*. Our data suggest that the variability of a *VOI* does not increase with *ICV*, which is also consistent with other published data. Therefore, we believe the nonlinear regression of Model 1 more closely reflects the noise mechanism underlying volumetric imaging measures.

There are some limitations to our study. First, our method was applied only on data processed by FreeSurfer. We believe the proposed nonlinear correction should be valid for data obtained from other neuroimaging software packages, but this remains to be seen. Second, the newly proposed method depends on model fitting, which is not as straightforward as the proportion method. We hope, however, that the power law formulation of Model 1 is clear enough for applied researchers to use with minimal burden. Third, the question of the reliability of the proposed power-proportion method has not been addressed (see, for example, Sanfilipo et al., 2004). Reliability will be examined in a follow-up study.

In conclusion, we proposed a power-proportion method for *ICV* correction in volumetric brain imaging analysis. The correction was based on the power law principle and motivated by the empirical evidence that volumes of many brain regions are not proportional to *ICV*. The new method was demonstrated to successfully remove the confounding effect of *ICV* using data from the PREDICT-HD study.

### Conflict of interest statement

The authors declare that the research was conducted in the absence of any commercial or financial relationships that could be construed as a potential conflict of interest.
